# Effect of incorporation of soy flour on functional, nutritional, and sensory properties of mushroom–moringa‐supplemented healthy soup

**DOI:** 10.1002/fsn3.594

**Published:** 2018-02-02

**Authors:** Suman Mohajan, Tania N. Orchy, Tasnim Farzana

**Affiliations:** ^1^ Institute of Food Science and Technology (IFST) Bangladesh Council of Scientific and Industrial Research (BCSIR) Dhaka Bangladesh

**Keywords:** functional properties, nutrition, sensory evaluation, soup, soy flour

## Abstract

The research study was conducted to evaluate the effect of soy flour on functional, nutritional, and sensory properties of mushroom–moringa‐supplemented soup which could be used as a protein‐supplemented ready‐to‐eat food. In this study, corn flour was supplemented with soy flour at different levels such as 20% (T4), 15% (T3), 10% (T2), and 5% (T1), and without soy flour was kept as control (T0). Fixed amount of mushroom and moringa leaf powder was added in all soup powders. Soup powders were analyzed for functional, nutritional, and sensory parameters. Bulk density (0.82–0.74 g/ml), dispersibility (82.1%–75.9%), pH (6.17–6.13), swelling capacity (3.98–3.65 ml/g), and viscosity were decreased, while water absorption capacity (70%–94%) was increased with increasing of soy flour percentages. Protein content of all the treatment groups increased from 10.66% to 19.97% along with a significant increased in fat (1.43%–6.97%), fiber (1.10%–2.30%), ash (15.77%–16.40%), and energy value (328.38–353.21 kcal/100 g) while decreased in moisture and carbohydrate content. On sensory evaluation, soup powders with 10% (T2) level of soy flour incorporation had highest scores for all the sensory attributes evaluated. Based on these results, it can be concluded that soy flour has effect on functional, nutritional, and sensory properties of soup powders and 10% supplementation of soy flour is suitable for ready‐to‐eat soup formulation. Besides these, use of mushroom and moringa leaf may also increase its nutritional value. Soup developed in this way may be sufficient to meet day‐to‐day nutritional requirements as a supplement.

## 1 INTRODUCTION

Urbanization and hectic life are closely related terms, and both of these two factors are now compelling people to be habituated with ready‐to‐eat processed junk foods which contain high sugar, fat, salt, and low nutrient value in terms of protein, fiber, vitamin, and mineral contents (Kaushik, Narang, & Parakh, 2011). Consumption of these nutrient‐deficient foods ultimately exaggerated the malnutrition‐related problems. To overcome these problems, we have to formulate ready‐to‐eat nutritious foods that are sufficient to fulfill daily requirement. In this regard, dry soup powder could be a well choice as its popularity is increasing day by day among different classes of people due to its short reconstitution time, protection from enzymatic and oxidative spoilage, and flavor stability at room temperature over long periods of time (6–12 months) (Rekha, Yadav, Dharmesh, Chauhan, & Ramteke, 2010). However, the locally available soup powders are not up to the mark in nutritional quality. The nutritional quality of traditional soup powders could be improved by supplementing it with mushroom, moringa leaf powders, and soy flour (Farzana & Mohajan, 2015; Wadud, Abid, Ara, Kosar, & Shah, 2004).

Mushrooms are of excellent source of complete protein (20%–40%), containing all the twenty essential amino acids, vitamins, dietary fibers, and minerals (Moharram, Salama, & Hussien, 2008). It is considered as an ideal food for diabetic patients due to its low calorie, fat, and carbohydrate. Mushroom is also an excellent source of vitamin B12 (Koyyalamudi, Jeong, Cho, & Pang, 2009) which is generally not present in plant foods and ideal choice for the vegetarians. Owing to this balanced status of protein, fat, carbohydrate, minerals, vitamins, amino acids, and active ingredients, mushroom can be used as a substitute of meat, fish, fruits, and vegetables (Kakon, Choudhury, & Saha, 2012).

*Moringa oleifera* is an excellent source of proteins, minerals, vitamins, antioxidants, tocopherols, and β‐carotene. It is also particularly rich in essential sulfur‐containing amino acids that are rarely found in daily diets (Ogunsina, Radha, & Singh, 2010). According to Fahey (2005), the nutritional quality of moringa leaf powder is superior to other available foods, for examples, vitamin C content of moringa leaves is seven times higher than that of oranges, vitamin A content is four times of carrots, potassium is three times of bananas, calcium is four times, and protein content is two times higher than that of milk. These nutritional benefits of *M. oleifera* make it an ideal choice for the preparation of various foods such as salads, soups, juices, and medicine.

Apart from these two plant sources, soybean, another nutrient‐dense plant source, could be an essential ingredient of functional foods as it is a good source of protein (45%) (Rahman, Saifullah, & Islam, 2012), containing most of essential amino acids (Nielsen, 1996), vitamins, and minerals. Soybean is also rich in lysine and tryptophan, two of the essential amino acids, which are limiting in cereals. Soybean protein is about four times of wheat, six times of rice grain, and it is also rich in Ca, P, and vitamins (A, B, C, and D) (Islam, Chowdhury, Islam, & Islam, 2007; Serrem, Kock, & Taylor, 2011). Soybean is also a good source of polyunsaturated fatty acids such as linoleic acid (approximately 50% of the total fat content) which is beneficial for health. These remarkable properties of soybean make it an ideal choice of supplementary foods such as soy‐supplemented bread, biscuits, and health drinks. (Mohamed, Rayas‐Duarte, Shogren, & Sessa, 2006; Taghdir et al., 2016).

Besides nutritional qualities, functional properties are also necessary to consider during food formulation because these properties reflect the complex interaction of food components with the nature of environment which are associated and measured (Siddiq, Nasir, Ravi, Dolan, & Butt, 2009; Singh, Kaur, Sodhi, & Khon, 2005). In case of starch‐based foods, physicochemical changes such as water absorption capacity, swelling capacity, viscosity, gel formation that occur during processing/cooking, and storage condition determine the foods quality and nutritional properties. These functional and nutritional qualities of foods have been found to be affected by soy flour (Taghdir et al., 2016). That is why, it is necessary to optimize the percentage of soybean in food supplementation.

In view of the above context, this study was designed to evaluate the effect of soy flour on the functional, nutritional, and sensory properties of mushroom–moringa‐supplemented soup.

## 2 MATERIALS AND METHODS

The study was carried out in the laboratory of Quality Control Research Section of Institute of Food Science and Technology (IFST), Bangladesh Council of Scientific and Industrial Research (BCSIR), Bangladesh.

### 2.1 Sample collection and processing

Soybean was collected from the Bangladesh Agricultural Research Institute. Oyster mushroom (*Pleurotus ostreatus*) was collected from the National Mushroom Development and Extension Center, Savar, Bangladesh. *M. oleifera* leaves were obtained from locally available moringa trees. Other ingredients were collected from the local market. Soybean seeds, mushrooms, and moringa leaves were processed according to the procedure described previously (Farzana, Mohajan, Saha, Hossain, & Haque, 2017).

### 2.2 Method of preparation of soup powder

**Figure 1:**
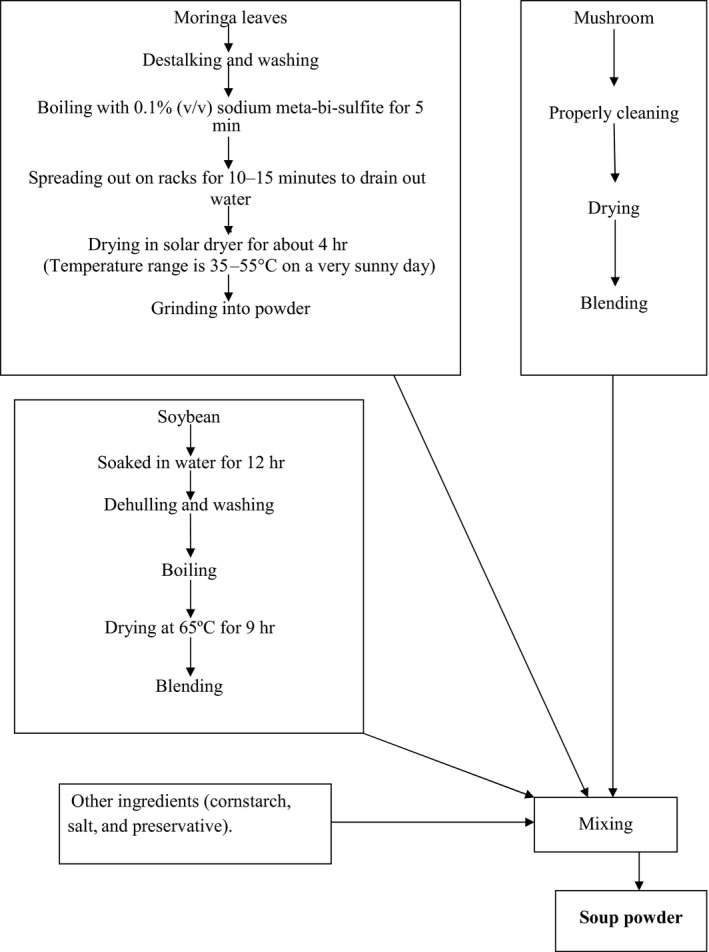
Flowchart for the preparation of soup powder

Corn flour was supplemented with soy flour in four proportions, 5%, 10%, 15%, and 20%, and designed as T1, T2, T3, and T4, respectively, whereas T0 (without soy flour) was kept as control. Fixed amount of mushroom (5%) and moringa leaf powder (6%) was added in all the treatments and control. The formulation and preparation of soup powder are shown in Table 1 and Figure 1, respectively. These newly developed soup powders were used for analysis of functional properties, nutritional, and sensory evaluation.

**Table 1:** Sample table of formulation of soup powders

Samples	Cornstarch (%)	Soy flour (%)	Mushroom (%)	Moringa leaf powder (%)	Salt (%)	Sodium benzoate (%)	Testing salt (%)
Control (T0)	68.475	–	5	6	12	0.025	8.5
T1 (5%)	63.475	5	5	6	12	0.025	8.5
T2 (10%)	58.475	10	5	6	12	0.025	8.5
T3 (15%)	53.475	15	5	6	12	0.025	8.5
T4 (20%)	48.475	20	5	6	12	0.025	8.5

### 2.3 Determination of functional properties of soup powders

The functional properties of soup powders to be determined includes, bulk density (determined using the method described by Onwuka, 2005), water absorption capacity (determined according to the method described by Onwuka, 2005), swelling capacity (using method of Bamidele, Ojedokun, & Fasogbon, 2015), dispersibility (determined using the method described by Kulkarni, Kulkarni, & Ingle, 1991), pH (using method of Mathew et al., 2015), and viscosity measurement (by modified method of Onwuka, 2005). For viscosity measurement, 5‐g portion of each of the soup powder samples was dissolved in 100 ml of water in a beaker and heated to boiling in a water bath. The beakers were then removed and cooled to room temperature (25°C). Each sample in the beakers was placed under the Brookfield DV‐E viscometer. Using spindle number 64 and speed of 20‐, 30‐, 50‐, 60‐, and 100‐rpm dial, readings were taken and recorded as centipoises (cp).

### 2.4 Proximate analysis of soup powders

The proximate composition of the soup powders were estimated according to the standard analytical methods (AOAC, 2005). The carbohydrate content was determined by calculated difference method, and the energy value was determined by multiplying the proportion of protein, fat, and carbohydrate by their respective physiological energy values and taking the sum of the products (Eneche, 1999; Farzana & Mohajan, 2015).

### 2.5 Sensory analysis

A trained sensory panelist of 10 members was selected for evaluating the sensory attributes including flavor, taste, texture, consistency, color, and overall acceptability using the 9‐point hedonic scale (Ranganna, 2000).

### 2.6 Statistical analysis

Statistical Package for the Social Sciences (SPSS version 15.0 SPSS Inc., Chicago, Illinois) was used for data analysis. Values were expressed as percentage and mean ± *SD*. One‐way ANOVA and Duncan test were used to determine the significance/nonsignificance. Means were separated using independent student *t* test.

## 3 RESULT AND DISCUSSION

### 3.1 Functional properties

The functional property is an important parameter for the suitability of diet behavior of nutrients in food during processing, storage, preparation, its application, and end use because it affects not only the general food quality but also its acceptability to the people (Adeleke & Odedeji, 2010). The results of functional properties of the soup powder samples are shown in Table 2.

**Table 2:** Functional properties of the soup powders with different levels of soy flour

Sample	Bulk density (g/ml)	Water absorption capacity (%)	Swelling capacity (ml/g)	Dispersibility (%)	pH	Viscosity (cp)				
						20 rpm (cp)	30 rpm (cp)	50 rpm (cp)	60 rpm (cp)	100 rpm (cp)
Control (T0)	0.82 ± 0.01^a^	70 ± 0.20^e^	3.98 ± 0.02^a^	82.1 ± 0.01^a^	6.17 ± 0.01^a^	1740 ± 5^a^	1060 ± 4^a^	700 ± 6^a^	580 ± 4^a^	390 ± 5^a^
T1 (5%)	0.80 ± 0.01^b^	75 ± 0.11^d^	3.88 ± 0.03^b^	80.0 ± 0.01^b^	6.16 ± 0.01^ab^	1065 ± 3^b^	750 ± 3^b^	540 ± 4^b^	450 ± 6^b^	297 ± 3^b^
T2 (10%)	0.78 ± 0.01^c^	83 ± 0.10^c^	3.80 ± 0.02^c^	78.6 ± 0.02^c^	6.15 ± 0.02^abc^	720 ± 4^c^	550 ± 5^c^	415 ± 5^c^	355 ± 5^c^	240 ± 4^c^
T3 (15%)	0.76 ± 0.01^d^	90 ± 0.20^b^	3.72 ± 0.04^d^	77 ± 0.03^d^	6.14 ± 0.01^bc^	585 ± 2^d^	450 ± 4^d^	315 ± 3^d^	265 ± 7^d^	174 ± 6^d^
T4 (20%)	0.74 ± 0.01^e^	94 ± 0.32^a^	3.65 ± 0.02^e^	75.9 ± 0.01^e^	6.13 ± 0.01^c^	150 ± 3^e^	100 ± 7^e^	80 ± 8^e^	70 ± 4^e^	62 ± 7^e^

Values are means of triplicates ± standard deviation. Values with the same superscript in a column are not significantly different (*p* > .05).

#### 3.1.1 Bulk density

The bulk density is important for dietary bulk and packaging requirements (Oppong, Arthur, Kwadwo, Badu, & Sakyi, 2015). Nutritionally, a loose bulk density not only promotes easy digestibility of food but also enhances nutrient and calorie density per feed that offers an extra advantage in formulating complementary foods (Osundahunsi & Aworh, 2002). In the present study, the bulk density of the soup powders ranged from 0.74 g/ml to 0.82 g/ml. The highest bulk density (0.82 g/ml) was found in the control (T0), while least (0.74 g/ml) was in the treatment T4 (20%). The bulk density of other treatments, T1 (5%), T2 (10%), and T3 (15%), was 0.80, 0.78 and 0.76 g/ml, respectively (Table 2). This decreasing trend of bulk density in the study is in agreement with the study of Akubor and Onimawo (2003). This may be due to the increasing proportion soy flour in the soup as soy flour has lower bulk density then corn flour (Akubor & Onimawo, 2003).

#### 3.1.2 Water absorption capacity

Water absorption capacity is the ability of product to incorporate water. The range of water absorption capacity of soup powders was 70%–94%. Treatment T4 (20%) had the highest water absorption capacity (94%) while the least (70%) in control (T0). The water absorption capacity for other treatments, T3 (15%), T2 (10%), and T1 (5%), was 90%, 83%, 75%, respectively (Table 2). This result is in consonant with the finding of Akubor and Onimawo (2003). The increase in water absorption capacity in the present study is due to the higher protein content of soy flour because proteins are capable of binding large quantities of water due to their ability to form hydrogen bonds between molecules and polar group on the polypeptide chain (Akubor & Onimawo, 2003). This property plays an influential role in bulking and consistency of ready‐to‐eat products (Niba, Bokanga, Jackson, Schlimme, & Li, 2001).

#### 3.1.3 Swelling capacity

The swelling capacity is the measure of the ability of starch to imbibe water and swell. In the present study, the swelling capacity of the five soup samples also followed the same pattern of bulk density, T0 > T1 (5%) > T2 (10%) > T3 (15%) > T4 (20%), such as control (T0) having the highest (3.98 ml/g) and treatments T4 (20%) having the lowest (3.65 ml/g) (Table 2). This decreasing trend of swelling capacity with the increasing of soy flour substitution is supported by a study of Julianti, Rusmarilin, Ridwansyah, & Yusraini, (2017). This may be as a result of lipids which act as amylose swelling inhibitor, starch and protein interaction, and attraction of their opposite's charges to form inclusion complexes during gelatinization which restricts swelling (Shimelis, Meaza, & Rakshit, 2006).

#### 3.1.4 Dispersibility

Dispersibility is an index that measures how well flour or flour blends can be rehydrated with water without formation of lumps. Dispersibility of the five soup powders ranged from 75.9% to 82.1%. Highest dispersibility (82.1%) was found in the control (T0) while least (75.9%) in the treatment T4 (20%). The dispersibility of other treatments, T1 (5%), T2 (10%), and T3 (15%), was 80%, 78.6%, and 77%, respectively (Table 2). This decreasing trend of dispersibility with the increasing of soy flour substitution is in agreement with the study of Olu et al. (2012). This may be due to increase in fat content with the increase in soy flour percentage in the soup formulation (Otegbayo, Samuel, & Alalade, 2013).

#### 3.1.5 pH

The pH of the five soup powders was ranged from 6.13 to 6.17, showing that the soup powders are slightly acidic in nature (Table 2). Highest pH was found in control (T0) (6.17) while least (6.13) in treatment T4 (20%). The decrease in pH in the present study is supported by a study of Novia, Juliyarsi, Melia, & Vermalida, (2013) where increased soy flour percentages decreased pH of the composite blend.

#### 3.1.6 Viscosity

Viscosity is an important characteristic of liquid foods in many areas of food processing. It gives an idea of the ability of a material to gel after cooking. There is a relationship between viscosity and shear rate. The viscosity of the five soup samples at different rpm is depicted in the Table 2. Viscosity of each treatments shows the same decreasing pattern with increasing rpm and supported by the study of Abdel‐Haleem and Omran (2014). Among the treatments, highest viscosity was observed for control (T0) and lowest for treatment T4 (20%). This decreasing trend may be due to decrease starch content with the increasing of soy flour and interaction of components such as fat and protein from soy flour with cornstarch that decrease the viscosity (Dautant, Simancas, Sandoval, & Muller, 2007; Julianti et al., 2017).

### 3.2 Proximate compositions of the developed soup powders (on dry basis)

#### 3.2.1 Moisture

In the present study, the moisture content of the five soup powders varied significantly and highest moisture content (2.83%) was found in control (T0), while lowest (1.71%) in treatment T4 (20%). The results showed that the moisture content gradually decreased from 2.83% to 1.71% with the increasing of soy flour (Table 3), supported by the findings of other studies (Banureka & Mahendran, 2009; Farzana & Mohajan, 2015). This may be explained as soy flour contained a greater amount of total dry solid with high emulsifying properties compared to other flours. According to El Wakeel (2007), moisture content less than 10% is considered as more proper for keeping quality of soup ingredients.

**Table 3:** Proximate analysis of the developed soup powders with different levels of soy flour (on dry basis)

Sample	Moisture (%)	Ash (%)	Protein (%)	Fat (%)	Fiber (%)	Carbohydrate (%)	Energy (kcal/100 g)
Control (T0)	2.83 ± 0.04^a^	15.77 ± 0.06^e^	10.66 ± 0.03^e^	1.43 ± 0.03^e^	1.10 ± 0.03^e^	68.21 ± 0.19^a^	328.38 ± 0.37^e^
T1 (5%)	2.51 ± 0.03^b^	15.91 ± 0.05^d^	12.57 ± 0.05^d^	2.76 ± 0.02^d^	1.24 ± 0.02^d^	65.01 ± 0.17^b^	335.16 ± 0.30^d^
T2 (10%)	2.30 ± 0.05^c^	16.01 ± 0.04^c^	15.54 ± 0.06^c^	4.10 ± 0.04^c^	1.56 ± 0.05^c^	60.49 ± 0.24^c^	341.02 ± 0.36^c^
T3 (15%)	1.92 ± 0.02^d^	16.19 ± 0.07^b^	17.50 ± 0.04^b^	5.69 ± 0.06^b^	1.75 ± 0.06^b^	56.95 ± 0.24^d^	348.99 ± 0.32^b^
T4 (20%)	1.71 ± 0.03^e^	16.40 ± 0.04^a^	19.97 ± 0.05^a^	6.97 ± 0.05^a^	2.30 ± 0.07^a^	52.65 ± 0.24^e^	353.21 ± 0.31^a^

Values are means of triplicates ± standard deviation. Values with the same superscript in a column are not significantly different (*p* > .05).

#### 3.2.2 Ash

The highest ash content (16.40%) was found in treatment T4 (20%), and lowest value (15.77%) was recorded in control (T0). In treatments T1 (5%), T2 (10%), and T3 (15%), the ash content was found to be 15.91%, 16.01%, and 16.19%, respectively (Table 3). This increasing trend of ash content with the increasing of soy flour is in agreement with other studies (Ayo, Ayo, Popoola, Omosebi, & Joseph, 2014; Farzana & Mohajan, 2015). Moreover, the ash content of the five soup samples is higher than the results of other studies (Rekha et al., 2010; Rubilar et al., 2012) due to the presence of soy flour, mushroom, and moringa in the soup preparation as soy flour, mushroom, and moringa leaves are good source of minerals, supported by other studies (Ayo et al., 2014; Sengev, Abu, & Gernah, 2013).

#### 3.2.3 Protein

In the present study, the protein content was found to increase from 10.66% to 19.97%. The highest protein content (19.97%) was found in treatment T4 (20%), and lowest (10.66%) was recorded for control (T0). In treatment groups, T1 (5%), T2 (10%), and T3 (15%), protein content was found to be 12.57%, 15.54%, and 17.50%, respectively (Table 3). This increasing trend of protein content is supported by other studies (Ayo et al., 2014; Banureka & Mahendran, 2009). This may be explained as soybean is a high‐protein legume and an excellent complement to lysine‐limited cereal protein (Garg, Malik, Lule, & Awasti, 2014). Due to high protein content, soy flour could be used as an economical protein supplement in biscuit, bread, pasta, and other cereal products. One important point in this study is that increased protein content was found not only in the treatment group but also in control as compared to other previous studies (Rahman et al., 2012; Rubilar et al., 2012). This may be due to soy flour, mushroom, and moringa leaf supplementation in the soup, supported by the other studies (Ayo et al., 2014; Farzana & Mohajan, 2015; Sengev et al., 2013). It could be assumed that addition of soy flour, mushroom, and moringa leaf in soup has a greater potential in overcoming protein‐calorie malnutrition in the world.

#### 3.2.4 Fat content

The fat content of the soup powders increased from 1.43% to 6.97% with the increase in soy flour. The highest fat content (6.97%) was found in treatment T4 (20%), and lowest (1.43%) was recorded for control (T0). In treatment groups, T1 (5%), T2 (10%), and T3 (15%), the fat content was found to be 2.76%, 4.10%, and 5.69%, respectively (Table 3). This increasing trend in fat content is in agreement with the other studies (Ayo et al., 2014; Banureka & Mahendran, 2009) on soy flour supplementation for the preparation of biscuits and could be explained as soy flour is globally considered as the number one edible oil source, containing 20%–24% of fat, most of which are unsaturated in nature, 61% polyunsaturated fat, and 24% monounsaturated fat (Reddy, 2004).

#### 3.2.5 Fiber content

The fiber content of the five soup powders significantly increased from 1.10% to 2.30%. The highest fiber content (2.30%) was found in treatment T4 (20%), and lowest (1.10%) was recorded for control (T0). In other treatment groups, T1 (5%), T2 (10%), and T3 (15%), the fiber content was found to be 1.24%, 1.56% and 1.75%, respectively (Table 3). Similar increasing trend in fiber content is also reported by other studies (Ayo et al., 2014; Farzana & Mohajan, 2015) on the supplementation of soy flour on the production of biscuits. This could be due to increase in soy flour in the blended soup mix (Ndife, Abdulraheem, & Zakari, 2011). This makes the soup powders a great choice of fiber.

#### 3.2.6 Carbohydrate content

Carbohydrate content was gradually decreased with the increase in supplementation of soy flour. Highest carbohydrate content (68.21%) was observed in control (T0), and lowest (52.65%) was recorded in treatment T4 (20%) (Table 3). Similar decreasing trend in carbohydrate content is also reported by other studies (Ayo et al., 2014; Banureka & Mahendran, 2009) and may be due to the low carbohydrate content of added soy flour (21%).

#### 3.2.7 Energy value

In the present study, the calorie content of the soup powders has been increased from 328.38 to 353.21 kcal/100 g with the addition of soy flour (Table 3). The increase in energy value may be due to the increase in protein and fat content with the increasing percentage of soy flour.

### 3.3 Sensory evaluation of soup powders

Sensory analysis of the soup powders is shown in Table 4. The taste is the primary factor which determines the acceptability of any product which has the highest impact as far as market success of product is concerned. The score for taste (8.6) was found highest in T2 (10%) and lowest (7.8) for T4 (20%). Soybean may have bitter taste at higher concentration. So, taste was found decreasing in other treatments T3 (15%) and T4 (20%). The mean scores for color of the soup powders changed from 7.9 to 8.5. The highest score (8.5) was obtained for treatment T2 (10%). From control (T0) to treatment T1 (5%) and T2 (10%), score for color was increasing and after then it was decreasing. The lowest score was obtained for T4 (20%). This may be due to decreasing color of cornstarch as with increasing soy flour percentages. In case of flavor of the five soup powders, the mean score was ranged from 8.0 to 8.4. Highest score (8.4) was obtained for T2 (10%), and after then, it was decreasing and lowest score (8.0) for T4 (20%). This may be due to beany odor of soybean. At higher concentration, it imparted its characteristics odor and resulted in low score for flavor (Akubor & Ukwuru, 2003). For texture, highest score (8.2) was obtained in T2 (10%) and lowest (7.8) in T4 (20%). It also followed the same trend as mean score for color, flavor, and taste. In case of consistency, the mean score was decreasing as increasing soy flour percentages. Highest score (8.5) was for control (T0) and lowest (7.6) for T4 (20%). This may be due to decreasing percentages of corn flour as starch imparted the consistency nature of soup powder. Overall acceptability includes many implications, which is an important parameter in organoleptic estimation. Treatment T2 (10%) had the highest mean value (8.3), while T4 (20%) had the least mean value (7.8) for the overall acceptability. The overall acceptability for control (T0) and T1 (5%) had a mean score of 8.2. At the 10% (T2) level of soy flour incorporation, the soup powder had highest scores for all the sensory attributes evaluated. Above this level, soup powder received a lower sensory score.

**Table 4:** Sensory attributes of soup powders with different levels of soy flour

Treatments	Taste	Color	Flavor	Texture	Consistency	Overall acceptability
Control (T0)	8.1 ± 0.01^d^	8.0 ± 0.01^d^	8.2 ± 0.02^c^	8.0 ± 0.02^c^	8.5 ± 0.03^a^	8.2 ± 0.02^b^
T1 (5%)	8.3 ± 0.02^b^	8.2 ± 0.02^b^	8.3 ± 0.03^b^	8.1 ± 0.01^b^	8.3 ± 0.01^b^	8.2 ± 0.01^b^
T2 (10%)	8.6 ± 0.03^a^	8.5 ± 0.03^a^	8.4 ± 0.01^a^	8.2 ± 0.02^a^	8.0 ± 0.02^c^	8.3 ± 0.01^a^
T3 (15%)	8.2 ± 0.01^c^	8.1 ± 0.01^c^	8.1 ± 0.02^d^	8.0 ± 0.03^c^	7.8 ± 0.01^d^	8.0 ± 0.02^c^
T4 (20%)	7.8 ± 0.02^e^	7.9 ± 0.02^e^	8.0 ± 0.01^e^	7.8 ± 0.02^d^	7.6 ± 0.02^e^	7.8 ± 0.03^d^

Values are expressed as means ± standard deviation. Values with the same superscript in a column are not significantly different (*p* > .05).

## 4 CONCLUSION

This study has demonstrated that soy flour addition to soup formulation had considerable effects on functional, nutritional, and sensory properties of mushroom–moringa soup. With the increase in soy flour percentage, the functional parameters such as bulk density, dispersibility, pH, swelling capacity, and viscosity were decreased, while water absorption capacity was increased. In case of nutritional properties, protein, fiber, ash, and fat contents were increased with the increase in soy flour percentages. On sensory evaluation, soup powders with 10% (T2) level of soy flour incorporation had highest scores for all the sensory attributes evaluated. Based on these results, it can be concluded that 10% soy flour incorporation is appropriate for developing ready‐to‐eat soup powder. These findings of the present study may help in developing commercial processing technology for effective utilization of soy flour especially in the manufacturing of soup.

## CONFLICT OF INTEREST

No conflict of interest.
